# Supplementary material for: Cost-effective micro-CT system for non-destructive testing of titanium 3D printed medical components
Source: PLoS One. 2022 Oct 10;17(10):e0275732. doi: 10.1371/journal.pone.0275732 (PMC9550031; doi:10.1371/journal.pone.0275732)
Supplement: Attachment — Submitted filename: Cobos 2022.docx [file pone.0275732.s001.docx]

Review

This paper presents a fantastic idea to make X-ray CT more accessible in the classroom and a number of other situations. However there are a number of problems which require significant revision to make this publishable. I hope the authors do take the time to make the proposed edits as I believe it to be useful to others in the community. A few core issues

- Some incorrect sweeping statements have been made. I have highlighted these where I can
- Some of the calibration parts could be better described.
- There are objects which contain dimensional measurements and are used to quantify the ability of the system. There is no evidence that these have been calibrated or measured. This needs looking at and have suggested below
- Please, please, please identify which commas are actually necessary

General Comments

“fluence” has been used instead of fluorescence throughout. Can you change assuming this was the intent. Its not a term I’ve come across in my time in tomography, and was unable to find a definition. Further many of the papers you cite us the term fluorescence so is the expected terminology of their readership.

There are far too many commas throughout the document to the point of being irritating. Please remove them as 75% of them are not required.

Often you slip into first person with “we” and “our”. Please remove

Abstract

Line 24 “The imaging performance of the system is characterized using a state-of-the-art, CT, quality-assurance phantom, and two titanium, 3D-printed, test specimens”

I didn’t like this sentence. “state of the art” is not true, they are printable and accessible by anyone. Instead could you replace with a sentence that describes the key features they test. E.g. The system was tested with a number of phantom containing X to evaluate Y. Arguably this sentence isn’t even necessary

Line 29 “the cost-effective….”

This sentence has a horrendous amount of commas. Please sort.

Line 30 “We believe…”

Make third person. First person not appropriate style, and correct throughout abstract

Introduction

I think there are a couple of useful review papers which can provide further context on how the technology of X-ray CT has developed over time. It can then be commented that while the technology has developed, cost has been forgone to maintain the greatest scientific potential. Reviews for this discussion would be:

- P J Withers et al (2021) X-ray Computed Tomography, Nature Reviews Method Primers 1(18)
- S R Stock (2008) Recent Advances in X-ray microtomography applied to Materials, International Materials Reviews 53(3)
- S R Stock (1999) X-ray microtomography of Materials, International Materials Reviews 44(4)

Another relevant review to include, specific because you mention additive manufacture

- A Thompson et al (2016) X-ray Computed Tomography for additive manufacturing: a review, Measurement Science and Technology

Maybe important because it talks about parameter selection, but does focus on the “speed” aspect

- E A Zwanenburg et al (2022) Review of high-speed imaging with lab-based x-ray computed tomography, Measurement Science and Technology

These would help strengthen the discussion. Also Withers and Zwanenburg would benefit your paper being newer publications as a number of yours are older e.g. your first couple are 2014, 2017, 2016, 2016.

Line 64 – I strongly disagree with your statement “in the case of medical components… spatial resolution needs to be on the order of 0.1mm”. It is very much dependent on the object and the context of what you wanted to observe! Glucose sensors for example which I have looked at needed 0.02mm. Acetabular hip cups achieved 0.03mm as per ref below, but it certainly would benefit from 0.01mm or better!

Kourra, Nadia, et al. "Computed tomography metrological examination of additive manufactured acetabular hip prosthesis cups." Additive Manufacturing 22 (2018): 146-152.

Line 78 – remove all first person and make third person

Methods

Throughout section A and B you use cm, can you change all to mm. It is standard in design to use mm or m and it’s a bit odd to have cm.

Line 88 remove “As previously mentioned”

Line 93 too much detail. Remove “the panels were secured…”

Line 95 similarly remove “this fabric was secured…”

Line 102 similarly, reword to “The camera-lens assembly was mounted to the frame with a sliding gantry plate that allowed camera-to-screen adjustments.”

Line 104 remove reference to clips. It’s a level of detail unnecessary

Section B

What hasn’t been made clear at any point is any shielding except for that covering the camera? Did you decide not to use any? Why? Please discuss this

Line 119 This sentence is a mess. Its too long and needs splitting and has far too many commas. The first three commas are the unnecessary ones

Line 127 the two commas are unnecessary

Line 128 “to match the exposure levels”… incorporate sentence on line 130 “note that…” into this sentence. Line 130 is unnecessary

Section C

Line 156 RGGB should be RGB? If not can you define this acronym. Also its something I am unfamiliar with going from RGB to greyscale so it would be useful to have a reference here.

Line 171 your distortion correction coupled with your FDK recon I am struggling with a bit. With your geometric distortion correction, I assume you are only correcting the issue arising from the camera tilt? If so please say. I assume this since you continue to use FDK for recon which is for circular cone beam. I just want to confirm that your geometrical distortion is not correcting the image effectively to a parallel beam in which case FDK is not the correct algorithm, its just simple FBP. But I think that would introduce more issues. Let me know what is going on here.

Line 175 the source alignment in this manner – do you have a reference for this method?

Line 177 “proper alignment of this ring was confirmed”. How? You’re right, its critical so how did you ensure that the ring was perfectly perpendicular to the direction of the source?

Line 188 your FDK reconstruction, for a 3104 width detector, you would need 4876 projections according to Nyquist. Its hence a surprise that you would use only 360 projections. How did this decision come about? Can you discuss? You say you then applied x3 binning (calculated going from 39 to 118 micron) because of the spatial resolution… in this case you effectively have a 1034 width detector which requires 1625 projections. It would be an explanation for your poor signal to noise.

I have a feeling this would have been done because of your limit on dose which is fine. Working backwards from Nyquist if you binned by 12 times instead you’ll get really nice reconstructions, albeit at low quality (472 microns). To resolve, I think it would be good to justify your # projections here, then in the discussion talk about additional shielding so you can increase the effective dose and therefore more projections.

Section D

Throughout this of the different phantoms, I am particularly nervous where you are using dimensional measurements to quantify how good your system is – there is no evidence of a traceable measurement to confirm this against. Have you measured any of them on CMM or SEM or anything? How do you know the volume of porosity in the gyroid to be exactly that (can do an Archimedes test)? The resolution phantom is really nice in concept, but 3D printing structures this small has some issues. Particularly a number of 100um voids that close together. You need to do something to measure these properly and state how this happened.

In the worse case you need to be upfront that none of these have been traceably measured and then redefine their purpose and the subsequent result.

- The porosity phantom. Given a design intent it has been 3D printed. Under this system operation we were able to identify pores as small as X, with an estimated porosity of Y
- The Ti6Al4V I really want a hi-res optical image of the surface with measurements as a minimum. Then you can say at the surface you know these measurements to be true. Then when things are not visible lower down it could be down to the system, or it could be a manufacturing error – you cannot confirm. Further where the line pairs do run further down you must state that you cannot guarantee their size below the surface. Another alternative which might be a nice idea is to scan it with a real X-ray CT system to serve as a gold standard. This would be much higher resolution and provide a sanity check. I am UK based so cannot suggest Canadian locations, but a number of US locations I know about are University Texas has an NSI system with Jessie Maisano where I am sure she would be happy to hear from you, or maybe University of Florida Ed Stanley/Stuart Stock.

Results

I think section A content is in the wrong place. I realise its an output of the idea which is why you’ve put it where you have, but I feel it should be back in the method with the system description because those choices were clearly already made.

The system design is part of the method, and the results are justifying that this works as you determined.

If not, your method for the system design needs to become less descript in terms of naming components and instead discuss characteristics of the specific components you want. Then Section A in results can cover your choices and why. I think this is messier, so advise you go with my earlier suggestion.

Line 293 “thanks to the use” language is too casual for a scientific paper. Infact I think a lot of section A has this sort of tone which could be improved.

Table 2 – right justify numbers. Also in the caption you should refer to the date these prices were obtained. This will change with time!

Line 303 – this is the only bit of part A which talks about a result. Given you are moving the rest to the method, I would suggest moving this paragraph into the discussion.

Section C b. needs some work and is confusing. I think what you are trying to say is that after your geometric distortion corrections, the reconstructed volume distances between beads were evaluated against the known values of the beads.

- How were the known values, known? CMM measured? To what accuracy? I doubt they were exactly 35mm, 24.75mm and 49.5mm…
- You jump between measurements of number of voxels and mm. that is confusing. Without going back and doing the maths myself I don’t know that 209 voxels is equal to a particular length we were after!
- It would be useful to have a graph of error vs test length, plotting each distance between the spheres. It should have error bars that represent the 95% confidence interval

Can you please give this short section a rework.

Section D See my comments above about know the precise porosity. The results of the resolution phantom comes across as “look at the figure” and you ran out of steam when writing. I think there is more detail you can put here about the success and not, and how this translates to the limitations of the system. This could be helped by referring to specific subfigues e.g. 16f) to motivate the discussion, and also demonstrate that the specific figures are actually useful.

Discussion

Even the first sentence has a ridiculous amount of commas!

Line 427 “we” remove all first person. Make third person

Line 471 This sentence is way too general and actually over reaching. The spatial resolution is one of the key benchmarks of micro-CT systems! So what benchmarks is it the same as?

In fact this entire paragraph is fraught with incorrect info. CMOS in commercial cameras DO have defects, the same as CT detectors. The have “bad pixel” corrections and “noisy pixel” corrections but these are buried in the camera software so you wouldn’t even know they are there. I think I would focus on the benchmarks you broadly say this system exceeds. Quantify numerically. Its probably sensible to quantify the decrease in ability against the cost i.e. this system costs 50x but the spatial resolution is about 10x worse than the highest possible resolution on typical 225kv micro ct scanners which is 5 um.

Line 459. You should easily be able to write a script to better calibrate this. You could do so in matlab and use the hough transform. Happy to accept the manual approach here but I think you can have a bit more detail on how this can be done.

Line 461 “in our experience” – not first person please! Change to third!

Line 495 When you talk about taking it to site I think it might be good to mention the size case this could theoretically be packed away in and the weight. It will give the idea to the reader of the degree of portability – can I carry in a backpack, or is this a heavier wheel case? Also what power source does it need?

Line 499 “as previously mentioned” – remove. I think this first sentence is duplicating what has just been said. Please review how this reads with the previous paragraph talking about archeological sites.

Conclusion

Line 531, 533 “we” remove,, line 537 “our”, make third person

Again the horrendous amount commas in this first sentence is infuriating.
